# Comparative analysis of disk diffusion and microscan versus broth microdilution methods for determining antimicrobial susceptibility of cefepime and piperacillin-tazobactam in *Escherichia coli* isolates carrying *bla*_OXA-1_

**DOI:** 10.1128/aac.00300-26

**Published:** 2026-06-22

**Authors:** Marta Carretero-Ledesma, Carlos Saúco-Carballo, Ana Velázquez Escudero, Inmaculada López-Hernández, Inés Portillo-Calderón, Lorena López-Cerero, José Manuel Rodríguez-Martínez, Jesús Rodríguez Baño, Álvaro Pascual, Fernando Docobo-Pérez

**Affiliations:** 1 Clinical Unit of Infectious Diseases and Microbiology, Virgen Macarena University Hospital 16582, Seville, Spain; 2 Institute of Biomedicine of Seville IBIS, Virgen Macarena University Hospital/CSIC/University of Seville, Seville, Spain; 3 Center for Biomedical Research Network on Infectious Diseases (CIBERINFEC), Madrid, Spain; 4 Department of Microbiology, Faculty of Medicine, University of Seville 16778, Seville, Spain; 5 Department of Medicine, Faculty of Medicine, University of Seville 16778, Seville, Spain; University of Pennsylvania Perelman School of Medicine, Philadelphia, Pennsylvania, USA

**Keywords:** *Escherichia coli*, *bla*_OXA-1_, antimicrobial susceptibility testing, disk diffusion, MicroScan, broth microdilution

## Abstract

Routine susceptibility testing often misses resistance in *bla*_OXA-1_-producing *E. coli*, risking therapeutic failure. Among 165 clinical isolates from the Spanish multicenter cohort, MicroScan and disk diffusion versus reference broth microdilution yielded suboptimal categorical agreement (73.9%–86.7%) for piperacillin-tazobactam (PTZ) and cefepime (FEP). MicroScan PTZ very major errors (30%) indicate resistance underestimation; disk diffusion PTZ major errors (23%) and both methods FEP major errors (8.3%–13.8%) indicate resistance overestimation. These errors justify cautious interpretation of routine susceptibility results.

## INTRODUCTION

OXA-1 (*bla*_OXA-1_) is a clinically relevant β-lactamase that compromises the activity of piperacillin-tazobactam (PTZ) and amoxicillin-clavulanic acid, increasing the use of carbapenems (1). *bla*_OXA-1_, alone or with extended-spectrum β-lactamases (ESBLs), increases PTZ minimum inhibitory concentrations (MICs) near clinical breakpoints, complicating interpretation and raising the risk of therapeutic failure (2, 3). Also, previous work from our group also demonstrated reduced cefepime (FEP) susceptibility in *bla*_OXA-1_-producing isolates (4), justifying FEP evaluation alongside PTZ to address AST performance gaps in *bla*_OXA-1_ producers. The MERINO trial revealed higher mortality in patients treated with PTZ for ESBL-producing Enterobacteriaceae, largely due to failures of routine methods, particularly disk diffusion (DD) and VITEK2, to detect PTZ resistance in *bla*_OXA-1_ carriers (1, 5). VITEK2 showed high major errors (ME) and very major errors (VMEs) rates in isolates with MICs 8–16 mg/L (1), and several studies have confirmed substantial variability of routine antimicrobial susceptibility testing (AST) methods compared with reference broth microdilution (BMD) (6, 7).

To the best of our knowledge, this study provides the first evaluation of MicroScan (MSC) analytical performance and DD versus reference BMD for PTZ and FEP in all 165 clinical *Escherichia coli* isolates (Table S1) carrying *bla*_OXA-1_ detected by whole-genome sequencing (WGS) from the multicentre PROBAC sepsis cohort (2016–2017) (8), in which *bla*_CTX-M-15_ was the most prevalent co-occurring β-lactamase (93/165, 56.3%), unlike prior studies limited to VITEK-2 (9), gradient strips (2), or DD (10) in smaller and mixes collection. AST followed ISO-20776-1 (11) using *E. coli* ATCC 25922 as QC and EUCAST v15.0 breakpoints (PTZ: S ≤ 8/>8 mg/L, ≥20/<20 mm; FEP: S ≤ 1/>4 mg/L, ≥27/<24 mm) (12, 13). BMD was performed in triplicate using CAMHB (Millipore, Merck), piperacillin (0.125–256 mg/L) plus tazobactam (4 mg/L) or FEP (0.008–16 mg/L) (Merck). MSC employed NegCombo 44 panels (Beckman Coulter, Spain) (dilution ranges for PTZ and FEP detailed in Tables 2 and 3) and DD 36-µg PTZ; 30-µg FEP discs (Oxoid, UK).

MICs and inhibition zone diameters obtained by MSC and DD were compared with modal MICs from BMD. Performance was evaluated using essential agreement (EA) and bias only for FEP, as its broader MIC range allows quantitative assessment, following ISO-20776-2:2021 criteria (14). PTZ was qualitatively analyzed due to limited MIC steps with MSC. Categorical agreement (CA), minor errors (mEs), ME, VME were defined and calculated using internationally established criteria for AST performance evaluation, integrating ISO and CLSI-M52 (15) standards as applied in recent EUCAST-based studies (16, 17) for both antibiotics. Acceptable thresholds were ≥90% EA and CA, ≤3% ME/VME, ≥95% sensitivity/specificity and bias within ±30%.

Overall, *E. coli* isolates carrying *bla*_OXA-1_ exhibited high levels of resistance: by BMD, PTZ MIC_50/90_ 16/>128 mg/L with 29% susceptibility, FEP MIC_50/90_ 16/>16 mg/L with 21.8% susceptibility (Table 1), and all MIC_90_ values exceeding the MSC detection limit. For FEP by BMD, MSC, and DD, resistance rates were 71%, 53%, and 69.1% for PTZ and 58.2%, 67.9%, and 63.6%, respectively. When comparing MSC with BMD (Tables 1 to 3), agreement within MIC ranges was 60% of isolates for PTZ and 66% for FEP, yielding CA of 75.2% and 73.9%, respectively. For FEP, MSC showed increased ME (13.8%), mE (21.8%), low specificity (61.1%), and bias of +40.5% for FEP and –30.0% for PTZ, indicating systematic overestimation of FEP MICs and underestimation of PTZ.

**TABLE 1:** Summary of MIC distribution, susceptibility category, categorical errors, sensitivity (SN), specificity (SP), categorical agreement (CA), and bias^*a*^^,^^*b*^

ATB	Methods	MIC distribution		Susceptibility category			Categorical errors			SN %	SP %	Ea %	CA %	Bias
		MIC_50_	MIC_90_	S *n* (%)	I *n* (%)	R *n* (%)	VME (%)	ME (%)	mE (%)					
PTZ	BMD	16/4	128/4	48 (29)	–	117 (71)	–	–	–	–	–	–	–	–
	MSC	16/4	>16/4	77 (47)	–	88 (53)	35 (30)	6 (12.5)	–	70.1	87.5	–	75.2	−30
	DD	–	–	51 (31)	–	114 (69.1)	14 (12)	11 (23)	–	88	77.1	–	84.8	
FEP	BMD	16	>16	36 (21.8)	33 (20)	96 (58.2)	–	–	–	–	–	**–**	–	–
	MSC	>8	>8	30 (18.2)	23 (13.9)	112 (67.9)	2 (2.1)	5 (13.8)	36 (21.8)	93.7	61.1	80	73.9	40.5
	DD	–	–	42 (25.5)	18 (11)	105 (63.6)	2 (2.1)	3 (8.3)	17 (10.3)	96.9	91.7	–	86.7	

^*a*^ Comparison of piperacillin-tazobactam (PTZ), and cefepime (FEP) susceptibility performed by Microscan (MSC) and disk diffusion (DD) against broth microdilution (BMD). Very major errors (VME), major errors (ME), minor error (mE).

^*b*^ –, not applicable.

**TABLE 2:** Results of the number of isolates at each MIC value for piperacillin-tazobactam obtained by broth microdilution and Microscan^*a*^

Piperacillin-tazobactam					
	MIC (mg/L) broth microdilution				
**MIC (mg/L) Microscan**		**≤8**	**16**	**>16**	**Total**
	**≤8**	42	**18**	**17**	77
	**16**	3	12	18	33
	**>16**	3	7	45	55
	**Total**	48	37	80	165

^*a*^ EUCAST established breakpoints were ≤8 mg/L for susceptibility and ≥16 mg/L for resistance; the gray area indicates categorical agreement (CA); the white area with bold numbers indicates very major errors (VMEs), and the white area with plain font corresponds to major errors (MEs).

**TABLE 3:** Results of the number of isolates at each MIC value for cefepime obtained by broth microdilution and Microscan^*a*^

Cefepime								
	MIC (mg/L) broth microdilution							
**MIC (mg/L) Microscan**		**≤0.5**	**1**	**2**	**4**	**8**	**>8**	**Total**
	**≤0.5**	16	0	*0*	*1*	**1**	**0**	18
	**1**	6	0	*4*	*2*	**1**	**0**	13
	**2**	*3*	*2*	6	2	*2*	*0*	15
	**4**	*3*	*1*	1	1	*1*	*1*	8
	**8**	4	0	*4*	*3*	1	0	12
	**>8**	1	0	*3*	*6*	4	85	99
	**Total**	33	3	18	15	10	86	165

^*a*^ EUCAST established breakpoints were ≤1 mg/L for susceptibility and ≥8 mg/L for resistance; the gray area indicates categorical agreement (CA), the white area with bold numbers indicates very major errors (VMEs); the numbers in italics correspond to minor errors (mEs), and the white area with plain font corresponds to major errors (MEs).

When comparing DD with BMD (Tables 1, 4 and 5), CA reached 84.8% and 86.7% for PTZ and FEP, respectively. However, DD produced high ME values (23% for PTZ; 8.3% for FEP) and reduced specificity (77.1% and 91.7%). Despite these discrepancies, DD for FEP maintained VME within acceptable limits and was overall more accurate than MSC, with ME: 13.8% and mE: 21.8%, although still below desirable analytical performance.

**TABLE 4:** Results of the number of isolates at each MIC value and inhibition zone diameter for piperacillin-tazobactam obtained by broth microdilution and disk diffusion, respectively^*a*^

Piperacillin-tazobactam																						
	Halo of inhibition (mm) disk diffusion																					
**MIC (mg/L) microdilution**		**0**	**7**	**8**	**9**	**10**	**11**	**12**	**13**	**14**	**15**	**16**	**17**	**18**	**19**	**20**	**21**	**22**	**23**	**26**	**27**	**28**
	**1**	0	0	0	0	0	0	0	0	0	0	0	0	0	0	0	0	0	0	1	1	0
	**2**	0	0	0	0	0	0	0	0	0	0	0	0	0	0	0	0	0	0	0	1	0
	**4**	0	0	0	0	0	0	0	0	0	0	0	0	0	1	1	3	3	0	0	0	1
	**8**	0	0	0	0	0	0	0	0	0	0	0	1	6	3	11	12	3	0	0	0	0
	**16**	2	0	0	0	0	0	0	1	1	2	3	8	5	10	**4**	**1**	**0**	**0**	**0**	**0**	**0**
	**32**	0	0	0	0	0	0	0	1	2	1	1	2	6	7	**2**	**2**	**1**	**0**	**0**	**0**	**0**
	**64**	0	0	0	1	0	0	2	1	6	5	2	0	3	1	**0**	**0**	**0**	**1**	**0**	**0**	**0**
	**128**	0	1	1	0	1	3	5	4	4	2	1	1	0	0	**1**	**1**	**0**	**0**	**0**	**0**	**0**
	**256**	0	0	0	0	0	0	0	1	2	1	1	0	0	0	**0**	**0**	**1**	**0**	**0**	**0**	**0**
	**>256**	1	0	0	0	0	1	0	0	0	0	0	0	0	0	**0**	**0**	**0**	**0**	**0**	**0**	**0**

^*a*^ EUCAST established breakpoints were ≤8 mg/L for susceptibility and ≥16 mg/L for resistance; the gray area indicates categorical agreement (CA); the white area with bold numbers indicates very major errors (VMEs), and the white area with plain font corresponds to major errors (MEs).

**TABLE 5:** Results of the number of isolates at each MIC value and inhibition zone diameter for cefepime obtained by broth microdilution and disk diffusion, respectively^*a*^

Cefepime																				
Halo of inhibition (mm) disk diffusion																				
**MIC (mg/L) microdilution**		**≤14**	**15**	**16**	**17**	**18**	**19**	**20**	**21**	**22**	**23**	**24**	**25**	**26**	**27**	**28**	**29**	**30**	**31**	**≥32**
	**0.03**	0	0	0	0	0	0	0	0	0	0	*0*	*0*	*0*	0	0	0	0	0	2
	**0.06**	0	0	0	0	0	0	0	0	0	0	*0*	*0*	*0*	0	0	0	1	0	1
	**0.12**	0	0	0	0	0	0	0	0	0	0	*0*	*0*	*0*	0	0	0	1	1	3
	**0.25**	0	0	0	0	0	0	0	0	0	0	*0*	*0*	*0*	1	0	2	1	4	4
	**0.5**	1	0	0	0	0	2	0	0	0	0	*0*	*0*	*0*	0	2	1	0	5	1
	**1**	0	0	0	0	0	0	0	0	0	0	*0*	*0*	*0*	1	0	0	1	1	0
	**2**	*0*	*0*	*0*	*0*	*1*	*0*	*0*	*1*	*2*	*0*	5	3	3	*0*	*2*	*1*	*0*	*0*	*0*
	**4**	*0*	*1*	*0*	*0*	*0*	*1*	*1*	*2*	*0*	*1*	1	3	2	*0*	*0*	*0*	*1*	*1*	*1*
	**8**	1	2	0	1	2	0	1	1	1	1	*1*	*0*	*0*	**0**	**1**	**0**	**0**	**0**	**0**
	**16**	1	1	0	0	1	2	0	0	0	0	*0*	*0*	*0*	**0**	**0**	**0**	**0**	**0**	**0**
	**≥32**	32	4	12	9	10	5	4	2	0	2	*0*	*0*	*0*	**0**	**0**	**0**	**0**	**0**	**1**

^*a*^ EUCAST established breakpoints were ≤1 mg/L for susceptibility and ≥8 mg/L for resistance; the gray area indicates categorical agreement (CA), the white area with bold numbers indicates very major errors (VMEs); the numbers in italics correspond to minor errors (mEs) and the white area with plain font corresponds to major errors (MEs).

In line with previous studies, the elevated MIC_50_ and MIC_90_ values for PTZ and FEP underscore the limited therapeutic usefulness of these antibiotics for infections caused by *bla*_OXA-1_-producing isolates (3, 4). Sensitivity and specificity analyses reinforced the limited reliability of both methods compared with BMD. MSC’s low sensitivity for PTZ (70.1%) shows difficulty detecting resistant isolates, while low specificity for FEP (61.1%) indicates inaccurate identification of susceptible strains. Overall, MSC tends to underestimate PTZ resistance, posing a risk of false susceptibility with potential impact on outcomes, whereas both MSC and DD tend to overestimate FEP resistance. These limitations are consistent with other reports that *bla*_OXA-1_ confers borderline PTZ MICs (8-16 mg/L), frequently misclassified by commercial systems, a behavior that reflects the complexity of PTZ resistance mediated by *bla*_OXA-1_, particularly within the EUCAST “area of technical uncertainty” (1, 3). In ATU, EUCAST recommends testing the isolate with an alternative method; in our setting, when this alternative method is DD, the still suboptimal performance and high ME rates (23%) for PTZ indicate that results should be interpreted cautiously. Laboratories may reasonably choose to report ATU results as resistant to avoid false susceptibility.

Recent studies have examined PTZ AST in Enterobacterales (9, 18, 19), refined *bla*_OXA-1_ detection strategies (2, 10), and shown that PTZ disk potency (CLSI 100/10 µg vs EUCAST 30/6 µg) influences disk diffusion performance (10). Concern over PTZ testing reliability largely originates from the MERINO trial, which found high VME rates with VITEK2 and an association between mortality and BMD MICs ≥ 32 mg/L (1). Additional evidence showed low probability of achieving PK/PD targets at MIC ≥16 mg/L, prompting CLSI to revise PTZ breakpoints and introduce the susceptible-dose dependent (SDD) category at MIC = 16 mg/L (20). Comparable studies described frequent categorical errors and poor CA values for PTZ with DD and gradient strip methods (2, 10), including DD overestimation of PTZ resistance in *bla*_OXA-1_*E. coli* with increased ME but acceptable VME (21). Our data extend these observations by providing the first MicroScan (MSC) evaluation in *bla*_OXA-1_ *E. coli*, showing substantial PTZ VME (30%) and supporting cautious interpretation of MSC results in this setting. For FEP, data remain scarce even in ESBL-producing enterobacteria (22). This study provides the first FEP evaluation specifically in *bla*_OXA-1_*E. coli*, confirming unsatisfactory CA in both methods with systematic resistance overestimation.

Our results are consistent with recent evidence highlighting important limitations of routine AST methods compared with the reference BMD when assessing PTZ and FEP activity in β-lactamase-producing Enterobacterales. In conclusion, this study underscores the need for caution when interpreting PTZ and FEP susceptibility results provided by MSC and DD in *E. coli* isolates carrying *bla*_OXA-1_. Since none of the methods fulfilled the performance criteria derived from international standards, including ISO recommendations for AST validation, with MSC showing the poorest performance and a clear risk of falsely categorizing resistant isolates as susceptible, particularly for PTZ. In this context, specific detection of *bla*_OXA-1_ in *E. coli* isolates is essential to accurately interpret the susceptibility to both antibiotics, minimize the risk of misclassifying resistant isolates, and support safer therapeutic decisions in clinical practice. Our study has limitations. The PROBAC cohort contained only *E. coli* isolates; future studies should evaluate *bla_OXA-1_*-producing *K. pneumoniae* and other Enterobacterales species.

## SUPPLEMENTAL MATERIAL

Table S1 aac.00300-26-s0001.pdf Detailed characterization of the PROBAC cohort of studied isolates.

ASM does not own the copyrights to Supplemental Material that may be linked to, or accessed through, an article. The authors have granted ASM a non-exclusive, world-wide license to publish the Supplemental Material files. Please contact the corresponding author directly for reuse.
