# Supplementary material for: Comparative analysis of disk diffusion and microscan versus broth microdilution methods for determining antimicrobial susceptibility of cefepime and piperacillin-tazobactam in Escherichia coli isolates carrying blaOXA-1
Source: Antimicrob Agents Chemother. 2026 Jun 22;70(8):e00300-26. doi: 10.1128/aac.00300-26 (PMC13436281; doi:10.1128/aac.00300-26)
Supplement: Supplementary file 1 [file aac.00300-26-s0001.pdf]

Supplementary Table S1: Detailed characterization of study isolates. *Escherichia coli* isolates carrying *bla* OXA-1 selected from the multicentre PROBAC cohort, from bacteraemia presenting sepsis. Selection was consecutive isolates, not restricted to first isolate per patient. The interpretation of the MIC results was performed according to the EUCAST guidelines. R = resistant, I = intermediate, S = susceptible, ATU= Area of Technical Uncertainty

Isolate	Hospital	Type of Hospital	Region	Source	ST	Phylogroup	Betalactamases	*bla* OXA-1 localization	IncF plasmid	Ampicillin	Amoxicillin/clavulanic acid	Ticarcillin	Piperacillin	Piperacillin/tazobactam	Cefazolin	Cefuroxime	Cefoxitin	Ceftazidime	Ceftazidime/clavulanic acid	Ceftazidime/avibactam	Cefotaxime	Cefotaxime/clavulanic acid	Cefepime	Ceftolozan/tazobactam	Cefixime	Aztreonam	Ertapenem	Imipenem	Meropenem	Gentamicin	Tobramycin	Amikacin	Nitrofurantoin	Norfloxacin	Ciprofloxacin	Levofloxacin	Tigecycline	Trimethoprim	Trimethoprim/sulfamethoxazole	Fosfomycin	Colistin	Mecillinam
CHJ108	Complejo Hospitalario Ciudad de Jaén	Tertiary-Care	Jaén (Andalucía)	Blood	131	B2	*bla* OXA-1, *bla* TEM-1B, *bla* CTX-M-15	Chromosome		>8 (R)	>32 (R)	>16 (R)	>16 (R)	<=8 (S)	>16 (R)	>8 (R)	<=8 (S)	>32 (R)	<=0.25	<=2 (S)	2 (R)	<=0.5	>8 (R)	<=1 (S)	>1 (R)	>4 (R)	<=0.12 (S)	<=1 (S)	<=0.12 (S)	>4 (R)	>4 (R)	<=8 (S)	<=64 (S)	>1 (R)	>1 (R)	1 (R)	<=1 (S)	<=2 (S)	<=2/38 (S)	32 (S)	<=2 (S)	<=8 (S)
CHJ119	Complejo Hospitalario Ciudad de Jaén	Tertiary-Care	Jaén (Andalucía)	Blood	2279	B2	*bla* OXA-1, *bla* CTX-M-15	Chromosome		>8 (R)	32 (R)	>16 (R)	>16 (R)	<=8 (S)	>16 (R)	>8 (R)	<=8 (S)	32 (R)	<=0.25	<=2 (S)	2 (R)	<=0.5	>8 (R)	<=1 (S)	>1 (R)	>4 (R)	<=0.12 (S)	<=1 (S)	<=0.12 (S)	<=2 (S)	>4 (R)	<=8 (S)	<=64 (S)	>1 (R)	>1 (R)	>1 (R)	<=1 (S)	<=2 (S)	<=2/38 (S)	<=8 (S)	<=2 (S)	<=8 (S)
CHJ125	Complejo Hospitalario Ciudad de Jaén	Tertiary-Care	Jaén (Andalucía)	Blood	131	B2	*bla* OXA-1, *bla* CTX-M-15	Chromosome		>8 (R)	32 (R)	>16 (R)	>16 (R)	<=8 (S)	>16 (R)	>8 (R)	<=8 (S)	32 (R)	<=0.25	<=2 (S)	>32 (R)	<=0.5	2 (I)	<=1 (S)	>1 (R)	>4 (R)	<=0.12 (S)	<=1 (S)	<=0.12 (S)	<=2 (S)	>4 (R)	<=8 (S)	<=64 (S)	>1 (R)	>1 (R)	>1 (R)	<=1 (S)	<=2 (S)	<=2/38 (S)	32 (S)	<=2 (S)	>8 (R)
CHJ128	Complejo Hospitalario Ciudad de Jaén	Tertiary-Care	Jaén (Andalucía)	Blood	1193	B2	*bla* OXA-1, *bla* TEM-1B, *bla* CTX-M-15	Plasmid	IncFIB(K)	>8 (R)	>32 (R)	>16 (R)	>16 (R)	>16 (R)	>16 (R)	>8 (R)	<=8 (S)	>32 (R)	<=0.25	<=2 (S)	>32 (R)	<=0.5	>8 (R)	<=1 (S)	>1 (R)	4 (I)	<=0.12 (S)	<=1 (S)	<=0.12 (S)	<=2 (S)	>4 (R)	16 (R)	<=64 (S)	>1 (R)	>1 (R)	>1 (R)	<=1 (S)	>4 (R)	>4/76 (R)	<=8 (S)	<=2 (S)	<=8 (S)
CHJ138	Complejo Hospitalario Ciudad de Jaén	Tertiary-Care	Jaén (Andalucía)	Blood	2279	B2	*bla* OXA-1, *bla* CTX-M-15	Chromosome		>8 (R)	16 (R)	>16 (R)	>16 (R)	<=8 (S)	>16 (R)	>8 (R)	<=8 (S)	8 (R)	<=0.25	<=2 (S)	2 (R)	<=0.5	>8 (R)	<=1 (S)	>1 (R)	4 (I)	<=0.12 (S)	<=1 (S)	<=0.12 (S)	<=2 (S)	>4 (R)	<=8 (S)	<=64 (S)	>1 (R)	>1 (R)	>1 (R)	<=1 (S)	<=2 (S)	<=2/38 (S)	<=8 (S)	<=2 (S)	<=8 (S)
CHJ33	Complejo Hospitalario Ciudad de Jaén	Tertiary-Care	Jaén (Andalucía)	Blood	131	B2	*bla* OXA-1, *bla* TEM-1B, *bla* CTX-M-15	Chromosome		>8 (R)	>32 (R)	>16 (R)	>16 (R)	<=8 (S)	>16 (R)	>8 (R)	<=8 (S)	>32 (R)	<=0.25	<=2 (S)	>32 (R)	<=0.5	>8 (R)	<=1 (S)	>1 (R)	4 (I)	<=0.12 (S)	<=1 (S)	<=0.12 (S)	>4 (R)	>4 (R)	<=8 (S)	<=64 (S)	>1 (R)	>1 (R)	>1 (R)	<=1 (S)	<=2 (S)	<=2/38 (S)	<=8 (S)	<=2 (S)	<=8 (S)
CHJ80	Complejo Hospitalario Ciudad de Jaén	Tertiary-Care	Jaén (Andalucía)	Blood	88	C	*bla* OXA-1	Chromosome		>8 (R)	>32 (R)	>16 (R)	>16 (R)	16 (R)	<=16 (S)	8 (S)	<=8 (S)	1 (S)	<=0.25	<=2 (S)	1 (S)	<=0.5	2 (I)	<=1 (S)	1 (R)	<=1 (S)	<=0.12 (S)	<=1 (S)	<=0.12 (S)	>4 (R)	>4 (R)	<=8 (S)	<=64 (S)	>1 (R)	>1 (R)	>1 (R)	<=1 (S)	>4 (R)	>4/76 (R)	<=8 (S)	<=2 (S)	<=8 (S)
CHJ83	Complejo Hospitalario Ciudad de Jaén	Tertiary-Care	Jaén (Andalucía)	Blood	131	B2	*bla* OXA-1, *bla* TEM-1B, *bla* CTX-M-15	Chromosome		>8 (R)	>32 (R)	>16 (R)	>16 (R)	<=8 (S)	>16 (R)	>8 (R)	<=8 (S)	>32 (R)	<=0.25	<=2 (S)	>32 (R)	<=0.5	>8 (R)	<=1 (S)	>1 (R)	>4 (R)	<=0.12 (S)	<=1 (S)	<=0.12 (S)	>4 (R)	>4 (R)	<=8 (S)	<=64 (S)	>1 (R)	>1 (R)	>1 (R)	<=1 (S)	<=2 (S)	<=2/38 (S)	<=8 (S)	<=2 (S)	<=8 (S)
CHJ92	Complejo Hospitalario Ciudad de Jaén	Tertiary-Care	Jaén (Andalucía)	Blood	131	B2	*bla* OXA-1, *bla* TEM-1B, *bla* CTX-M-15	Chromosome		>8 (R)	>32 (R)	>16 (R)	>16 (R)	<=8 (S)	>16 (R)	>8 (R)	<=8 (S)	32 (R)	<=0.25	<=2 (S)	>32 (R)	<=0.5	>8 (R)	<=1 (S)	>1 (R)	>4 (R)	<=0.12 (S)	<=1 (S)	<=0.12 (S)	>4 (R)	>4 (R)	<=8 (S)	<=64 (S)	>1 (R)	>1 (R)	>1 (R)	<=1 (S)	<=2 (S)	<=2/38 (S)	<=8 (S)	<=2 (S)	>8 (R)
CHJ93	Complejo Hospitalario Ciudad de Jaén	Tertiary-Care	Jaén (Andalucía)	Blood	131	B2	*bla* OXA-1, *bla* TEM-1A	Plasmid	IncFII	>8 (R)	>32 (R)	>16 (R)	>16 (R)	16 (R)	16 (R)	<=4 (S)	<=8 (S)	1 (S)	<=0.25	<=2 (S)	1 (S)	<=0.5	4 (I)	<=1 (S)	1 (R)	<=1 (S)	<=0.12 (S)	<=1 (S)	<=0.12 (S)	<=2 (S)	>4 (R)	<=8 (S)	<=64 (S)	>1 (R)	>1 (R)	>1 (R)	<=1 (S)	<=2 (S)	<=2/38 (S)	<=8 (S)	<=2 (S)	<=8 (S)
HCS4402	Hospital Regional Universitario de Málaga	Tertiary-Care	Málaga (Andalucía)	Blood	131	B2	*bla* OXA-1, *bla* CTX-M-15	Chromosome		>8 (R)	32 (R)	>16 (R)	>16 (R)	<=8 (S)	>16 (R)	>8 (R)	<=8 (S)	32 (R)	<=0.25	<=2 (S)	>32 (R)	<=0.5	>8 (R)	<=1 (S)	>1 (R)	>4 (R)	<=0.12 (S)	<=1 (S)	<=0.12 (S)	>4 (R)	>4 (R)	<=8 (S)	<=64 (S)	>1 (R)	>1 (R)	>1 (R)	<=1 (S)	>4 (R)	>4/76 (R)	<=8 (S)	<=2 (S)	>8 (R)
HCS4933	Hospital Regional Universitario de Málaga	Tertiary-Care	Málaga (Andalucía)	Blood	131	B2	*bla* OXA-1, *bla* CTX-M-15	Chromosome		>8 (R)	32 (R)	>16 (R)	>16 (R)	<=8 (S)	>16 (R)	>8 (R)	<=8 (S)	32 (R)	<=0.25	<=2 (S)	>32 (R)	<=0.5	>8 (R)	<=1 (S)	>1 (R)	>4 (R)	<=0.12 (S)	<=1 (S)	<=0.12 (S)	<=2 (S)	>4 (R)	<=8 (S)	<=64 (S)	>1 (R)	>1 (R)	>1 (R)	<=1 (S)	<=2 (S)	<=2/38 (S)	<=8 (S)	<=2 (S)	>8 (R)
HCS4959	Hospital Regional Universitario de Málaga	Tertiary-Care	Málaga (Andalucía)	Blood	131	B2	*bla* OXA-1, *bla* CTX-M-15	Chromosome		>8 (R)	32 (R)	>16 (R)	>16 (R)	<=8 (S)	>16 (R)	>8 (R)	<=8 (S)	>32 (R)	<=0.25	<=2 (S)	2 (R)	<=0.5	>8 (R)	<=1 (S)	>1 (R)	>4 (R)	<=0.12 (S)	<=1 (S)	<=0.12 (S)	<=2 (S)	>4 (R)	<=8 (S)	<=64 (S)	>1 (R)	>1 (R)	>1 (R)	<=1 (S)	>4 (R)	>4/76 (R)	<=8 (S)	<=2 (S)	<=8 (S)
HCS92	Hospital Regional Universitario de Málaga	Tertiary-Care	Málaga (Andalucía)	Blood	131	B2	*bla* OXA-1, *bla* TEM-1C, *bla* CTX-M-15	Chromosome		>8 (R)	32 (R)	>16 (R)	>16 (R)	>16 (R)	>16 (R)	>8 (R)	<=8 (S)	2 (R)	<=0.25	<=2 (S)	>32 (R)	<=0.5	>8 (R)	<=1 (S)	>1 (R)	>4 (R)	<=0.12 (S)	<=1 (S)	<=0.12 (S)	<=2 (S)	>4 (R)	<=8 (S)	<=64 (S)	>1 (R)	>1 (R)	>1 (R)	<=1 (S)	<=2 (S)	<=2/38 (S)	<=8 (S)	<=2 (S)	>8 (R)
HJF5651	Jerez de la Frontera Hospital	Tertiary-Care	Jerez de la Frontera, Cádiz (Andalucía)	Blood	131	B2	*bla* OXA-1, *bla* CTX-M-15	Chromosome		>8 (R)	>32 (R)	>16 (R)	>16 (R)	<=8 (S)	>16 (R)	>8 (R)	<=8 (S)	32 (R)	<=0.25	<=2 (S)	>32 (R)	<=0.5	>8 (R)	<=1 (S)	>1 (R)	4 (I)	<=0.12 (S)	<=1 (S)	<=0.12 (S)	<=2 (S)	>4 (R)	<=8 (S)	<=64 (S)	>1 (R)	>1 (R)	>1 (R)	<=1 (S)	>4 (R)	>4/76 (R)	<=8 (S)	<=2 (S)	>8 (R)
HJF6410	Jerez de la Frontera Hospital	Tertiary-Care	Jerez de la Frontera, Cádiz (Andalucía)	Blood	88	C	*bla* OXA-1	Chromosome		>8 (R)	>32 (R)	>16 (R)	>16 (R)	>16 (R)	>16 (R)	>8 (R)	<=8 (S)	1 (S)	<=0.25	<=2 (S)	1 (S)	<=0.5	2 (I)	<=1 (S)	1 (R)	<=1 (S)	<=0.12 (S)	<=1 (S)	<=0.12 (S)	<=2 (S)	<=2 (S)	<=8 (S)	<=64 (S)	>1 (R)	>1 (R)	>1 (R)	<=1 (S)	>4 (R)	>4/76 (R)	<=8 (S)	<=2 (S)	<=8 (S)
HJF6420	Jerez de la Frontera Hospital	Tertiary-Care	Jerez de la Frontera, Cádiz (Andalucía)	Blood	131	B2	*bla* OXA-1, *bla* TEM-1B, *bla* CTX-M-15	Chromosome		>8 (R)	>32 (R)	>16 (R)	>16 (R)	<=8 (S)	>16 (R)	>8 (R)	<=8 (S)	16 (R)	<=0.25	<=2 (S)	32 (R)	<=0.5	>8 (R)	<=1 (S)	>1 (R)	>4 (R)	<=0.12 (S)	<=1 (S)	<=0.12 (S)	>4 (R)	>4 (R)	<=8 (S)	<=64 (S)	>1 (R)	>1 (R)	>1 (R)	<=1 (S)	<=2 (S)	<=2/38 (S)	<=8 (S)	<=2 (S)	<=8 (S)
HUCH2877	Hospital Regional Universitario de Málaga	Tertiary-Care	Málaga (Andalucía)	Blood	131	B2	*bla* OXA-1, *bla* TEM-1B, *bla* CTX-M-15	Plasmid	IncFIA	>8 (R)	>32 (R)	>16 (R)	>16 (R)	>16 (R)	>16 (R)	>8 (R)	<=8 (S)	32 (R)	<=0.25	<=2 (S)	>32 (R)	<=0.5	>8 (R)	2 (S)	>1 (R)	4 (I)	<=0.12 (S)	<=1 (S)	<=0.12 (S)	<=2 (S)	>4 (R)	>16 (R)	<=64 (S)	>1 (R)	>1 (R)	>1 (R)	<=1 (S)	<=2 (S)	<=2/38 (S)	<=8 (S)	<=2 (S)	<=8 (S)
HUCH4223	Hospital Regional Universitario de Málaga	Tertiary-Care	Málaga (Andalucía)	Blood	648	F	*bla* OXA-1	Plasmid	IncFII(pRSB107)	>8 (R)	>32 (R)	>16 (R)	>16 (R)	16 (R)	16 (S)	>8 (R)	16 (R)	1 (S)	<=0.25	<=2 (S)	2 (R)	<=0.5	4 (I)	<=1 (S)	>1 (R)	<=1 (S)	<=0.12 (S)	<=1 (S)	<=0.12 (S)	<=2 (S)	>4 (R)	16 (R)	<=64 (S)	>1 (R)	>1 (R)	>1 (R)	<=1 (S)	>4 (R)	>4/76 (R)	<=8 (S)	<=2 (S)	<=8 (S)
HUCH4298	Hospital Regional Universitario de Málaga	Tertiary-Care	Málaga (Andalucía)	Blood	88	C	*bla* OXA-1	Chromosome		>8 (R)	>32 (R)	>16 (R)	>16 (R)	>16 (R)	16 (S)	>8 (R)	<=8 (S)	1 (S)	<=0.25	<=2 (S)	4 (R)	<=0.5	>8 (R)	<=1 (S)	1 (R)	<=1 (S)	<=0.12 (S)	<=1 (S)	<=0.12 (S)	>4 (R)	>4 (R)	<=8 (S)	<=64 (S)	>1 (R)	>1 (R)	>1 (R)	<=1 (S)	>4 (R)	>4/76 (R)	<=8 (S)	<=2 (S)	<=8 (S)
HUCH4634	Hospital Regional Universitario de Málaga	Tertiary-Care	Málaga (Andalucía)	Blood	410	C	*bla* OXA-1, *bla* CTX-M-15	Plasmid	IncFIA	>8 (R)	>32 (R)	>16 (R)	>16 (R)	<=8 (S)	>16 (R)	>8 (R)	<=8 (S)	32 (R)	<=0.25	<=2 (S)	32 (R)	<=0.5	>8 (R)	<=1 (S)	>1 (R)	4 (I)	<=0.12 (S)	<=1 (S)	<=0.12 (S)	>4 (R)	>4 (R)	<=8 (S)	<=64 (S)	>1 (R)	>1 (R)	>1 (R)	<=1 (S)	<=2 (S)	<=2/38 (S)	>64 (R)	<=2 (S)	>8 (R)
HUCH4762	Hospital Regional Universitario de Málaga	Tertiary-Care	Málaga (Andalucía)	Blood	88	C	*bla* OXA-1	Chromosome		>8 (R)	>32 (R)	>16 (R)	>16 (R)	>16 (R)	16 (S)	>8 (R)	<=8 (S)	1 (S)	<=0.25	<=2 (S)	8 (R)	<=0.5	>8 (R)	<=1 (S)	1 (R)	<=1 (S)	<=0.12 (S)	<=1 (S)	<=0.12 (S)	<=2 (S)	<=2 (S)	<=8 (S)	<=64 (S)	>1 (R)	>1 (R)	>1 (R)	<=1 (S)	>4 (R)	>4/76 (R)	<=8 (S)	<=2 (S)	>8 (R)
HUPM1632	Hospital Universitario Puerta del Mar	Tertiary-Care	Cádiz (Andalucía)	Blood	131	B2	*bla* OXA-1, *bla* CTX-M-15	Plasmid	IncFIA	>8 (R)	>32 (R)	>16 (R)	>16 (R)	16 (R)	>16 (R)	>8 (R)	<=8 (S)	32 (R)	<=0.25	<=2 (S)	>32 (R)	<=0.5	>8 (R)	<=1 (S)	>1 (R)	4 (I)	<=0.12 (S)	<=1 (S)	<=0.12 (S)	>4 (R)	>4 (R)	16 (R)	<=64 (S)	>1 (R)	>1 (R)	>1 (R)	<=1 (S)	>4 (R)	>4/76 (R)	<=8 (S)	<=2 (S)	<=8 (S)
HUPM5513	Hospital Universitario Puerta del Mar	Tertiary-Care	Cádiz (Andalucía)	Blood	131	B2	*bla* OXA-1, *bla* CTX-M-15	Plasmid	IncFII	>8 (R)	>32 (R)	>16 (R)	>16 (R)	>16 (R)	16 (S)	>8 (R)	<=8 (S)	32 (R)	<=0.25	<=2 (S)	>32 (R)	<=0.5	>8 (R)	<=1 (S)	>1 (R)	4 (I)	<=0.12 (S)	<=1 (S)	<=0.12 (S)	>4 (R)	>4 (R)	16 (R)	<=64 (S)	>1 (R)	>1 (R)	>1 (R)	<=1 (S)	>4 (R)	>4/76 (R)	<=8 (S)	<=2 (S)	<=8 (S)
HUPM5530	Hospital Universitario Puerta del Mar	Tertiary-Care	Cádiz (Andalucía)	Blood	10	A	*bla* OXA-1, *bla* CTX-M-15	Plasmid	IncFII(pRSB107)	>8 (R)	>32 (R)	>16 (R)	>16 (R)	>16 (R)	>16 (R)	>8 (R)	<=8 (S)	>32 (R)	<=0.25	<=2 (S)	>32 (R)	<=0.5	>8 (R)	<=1 (S)	>1 (R)	4 (I)	<=0.12 (S)	<=1 (S)	<=0.12 (S)	<=2 (S)	>4 (R)	<=8 (S)	<=64 (S)	<=0.5 (S)	<=0.06 (S)	<=0.5 (S)	<=1 (S)	<=2 (S)	<=2/38 (S)	<=8 (S)	<=2 (S)	<=8 (S)
HUPM5581	Hospital Universitario Puerta del Mar	Tertiary-Care	Cádiz (Andalucía)	Blood	410	C	*bla* OXA-1, *bla* CTX-M-15	Plasmid	IncFIA	>8 (R)	>32 (R)	>16 (R)	>16 (R)	<=8 (S)	>16 (R)	>8 (R)	<=8 (S)	>32 (R)	<=0.25	<=2 (S)	>32 (R)	<=0.5	>8 (R)	<=1 (S)	>1 (R)	4 (I)	<=0.12 (S)	<=1 (S)	<=0.12 (S)	<=2 (S)	>4 (R)	<=8 (S)	<=64 (S)	>1 (R)	>1 (R)	>1 (R)	<=1 (S)	<=2 (S)	<=2/38 (S)	<=8 (S)	<=2 (S)	<=8 (S)
HUPM5840	Hospital Universitario Puerta del Mar	Tertiary-Care	Cádiz (Andalucía)	Blood	131	B2	*bla* OXA-1	Chromosome		>8 (R)	32 (R)	>16 (R)	>16 (R)	<=8 (S)	16 (S)	<=4 (S)	<=8 (S)	1 (S)	<=0.25	<=2 (S)	1 (S)	<=0.5	<=0.5 (S)	<=1 (S)	1 (R)	<=1 (S)	<=0.12 (S)	<=1 (S)	<=0.12 (S)	<=2 (S)	>4 (R)	<=8 (S)	<=64 (S)	>1 (R)	>1 (R)	>1 (R)	<=1 (S)	<=2 (S)	<=2/38 (S)	<=8 (S)	<=2 (S)	<=8 (S)
